# Objective Data From Patients With Persistent Spinal Pain Syndrome Type II Implanted With Spinal Cord Stimulator

**DOI:** 10.1111/papr.70093

**Published:** 2025-10-16

**Authors:** Alaa Abd‐Elsayed, Christopher Gilligan

**Affiliations:** ^1^ Department of Anesthesiology University of Wisconsin School of Medicine and Public Health Madison Wisconsin USA; ^2^ Robert Wood Johnson University Hospital New Brunswick New Jersey USA

Persistent spinal pain syndrome Type 2 (PSPS Type 2) causes severe pain associated with limitations of physical activities and affects the emotional and social aspects of patients' lives.

A study by Hamm‐Faber et al. [1] conducted a study to investigate the feasibility of collecting objective data from an activity tracker and a neurostimulator device to evaluate physical activity. The authors also evaluated the experiences of patients and healthcare professionals.

The authors concluded that the objective collection of data is feasible with a neuromodulation device and an activity tracker if the watch is correctly worn on the wrist or with clear instructions.

We encourage readers to read the full manuscript published in *Pain Practice*.

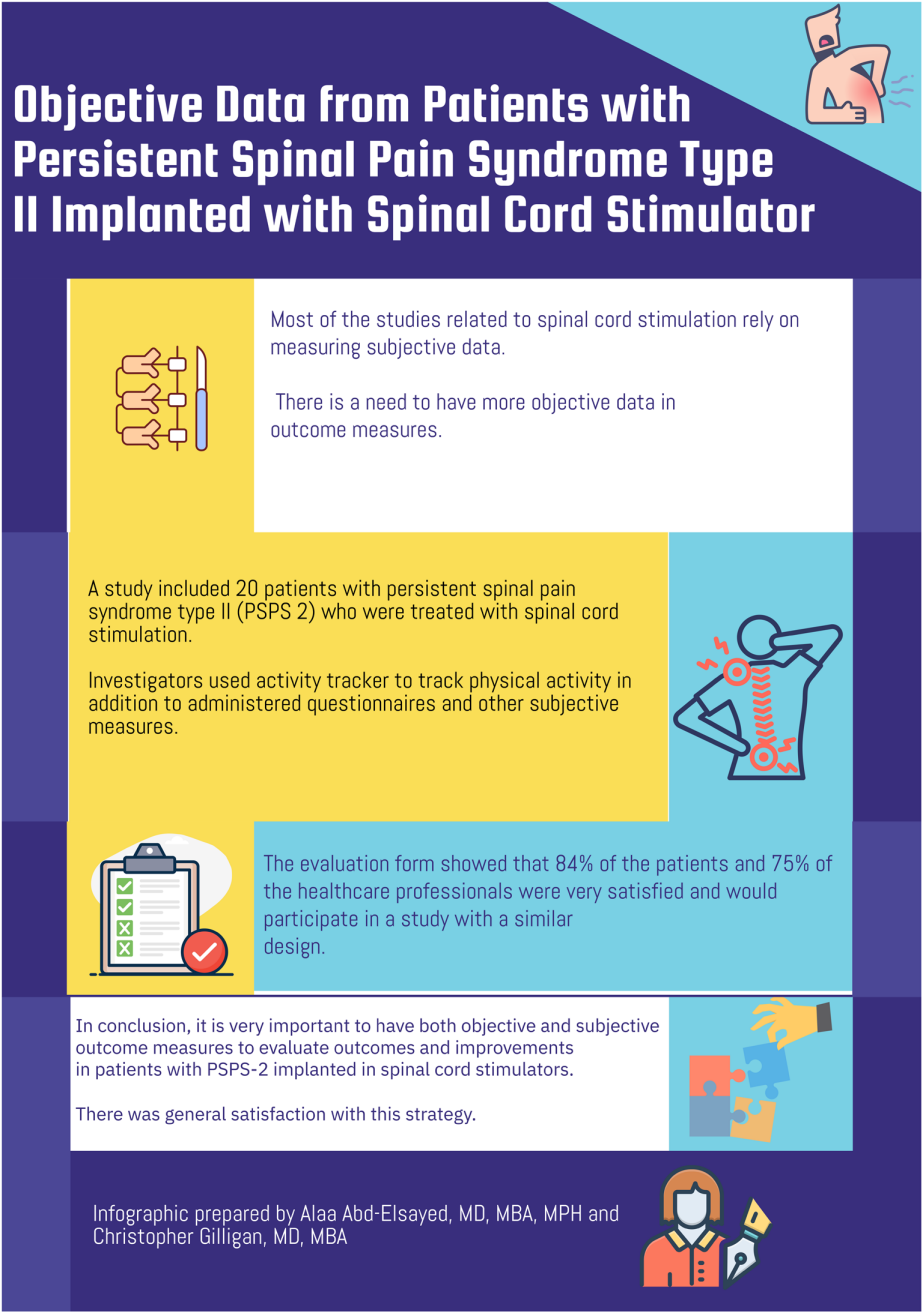

## Conflicts of Interest

Dr. Christopher Gilligan is the editor in chief of *Pain Practice* and Dr. Alaa Abd‐Elsayed is a section editor of *Pain Practice*.

## Data Availability

The authors have nothing to report.
